# Chronic pain as a state-constrained brain network disorder: a dynamical systems model integrating physiological regulation and self-organisation

**DOI:** 10.3389/fpain.2026.1825571

**Published:** 2026-06-22

**Authors:** Tim Ho, Mark Ryan

**Affiliations:** 1 Sydney Medical School, Westmead Clinical School, Faculty of Medicine and Health, The University of Sydney, Sydney, NSW, Australia; 2 Cingulum Health, Sydney, NSW, Australia

**Keywords:** autonomic regulation, brain network dynamics, chronic pain, neuromodulation, salience network, triple-network model

## Abstract

Chronic pain is increasingly understood as a disorder of large-scale brain network organisation rather than a direct consequence of persistent nociceptive input. Neuroimaging studies have consistently identified alterations in the salience network (SN), default mode network (DMN), and central executive network (CEN), suggesting that persistent pain involves disturbances in the dynamic coordination of networks that integrate bodily signals, cognition, and self-related processing. However, prevailing models largely interpret these findings as intrinsic network dysfunction and provide limited explanation for the persistence and variability of pain states. In this narrative review, we propose a state-constrained triple-network model of self-organisation in which interactions among the SN, DMN, and CEN are continuously shaped by organism-level physiological regulation, including sleep organisation, autonomic balance, and interoceptive signalling. Within this framework, chronic pain may emerge when disturbances in physiological regulation bias salience processing toward bodily threat signals and stabilise network configurations that reinforce self-referential pain processing. We further interpret these dynamics within a dynamical systems framework in which persistent pain reflects a stable attractor configuration of triple-network interactions maintained by physiological regulatory constraints. Neuromodulatory interventions such as repetitive transcranial magnetic stimulation (rTMS) may act as perturbations capable of increasing network flexibility, while durable recovery may require restoration of physiological regulation and experiential integration. This framework links network neuroscience, physiological regulation, and the phenomenology of self-experience, providing a multilevel model for understanding the persistence of chronic pain and informing future approaches to integrated treatment.

## Introduction

Chronic pain is increasingly recognised as a disorder of brain network organisation rather than a simple consequence of persistent nociceptive input (1, 2). Neuroimaging studies have consistently demonstrated alterations in large-scale brain networks in individuals with chronic pain, particularly involving the salience network (SN), default mode network (DMN), and central executive network (CEN) (3, 4). These networks form the basis of the triple-network model, which has been proposed as a unifying framework for understanding a range of neuropsychiatric conditions (5). Within this architecture, the SN detects and prioritises behaviourally relevant internal and external stimuli, the DMN supports autobiographical and self-referential processing, and the CEN enables cognitive control and goal-directed behaviour (5, 6).

Evidence from chronic pain research suggests that persistent pain is associated with increased SN activity, altered DMN connectivity, and impaired executive regulation (7, 3, 2). These findings have led to the proposal that chronic pain represents a disorder of large-scale network dynamics (1). However, current models largely interpret these changes as intrinsic network dysfunction and provide limited explanation for the persistence and variability of pain states (2).

Importantly, chronic pain is also consistently associated with disturbances of physiological regulation, including disrupted sleep architecture, autonomic imbalance, and altered interoceptive signalling (8, 9). These organism-level regulatory processes are known to influence brain network dynamics (10, 11) but remain largely absent from prevailing models of chronic pain.

Here we propose a state-constrained triple-network model of self-organisation, in which interactions among the SN, DMN, and CEN are shaped by physiological regulatory states. Within this framework, chronic pain may emerge from the interaction of physiological regulation, large-scale brain network dynamics, and experiential processes over time, producing state-dependent transitions between patterns of large-scale brain network organisation. The model does not propose physiological dysregulation as a single cause of chronic pain, but as part of the regulatory context that may shape how pain-related network states emerge, persist, or fluctuate over time. From a dynamical systems perspective, these interactions can be understood as state-constrained self-organisation within interacting large-scale brain networks, linking network neuroscience with clinical phenomenology and suggesting new directions for multimodal treatment strategies.

The model’s main contribution is to place established SN, DMN, and CEN alterations in chronic pain within a broader physiological regulatory context that may shape the stability and flexibility of pain-related network states over time.

**Figure 1:**
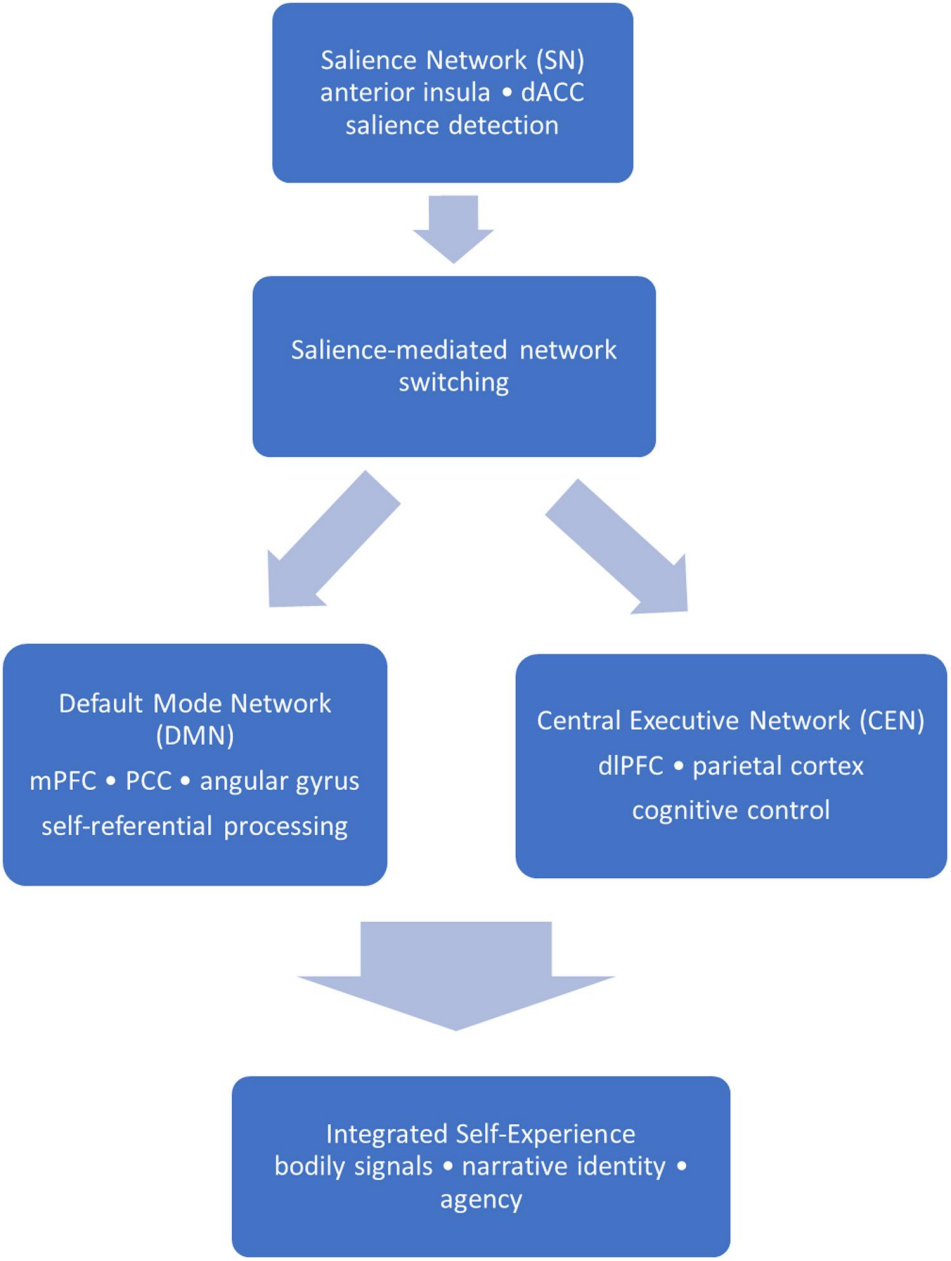
Triple-network architecture supporting self-organisation. The triple-network model describes coordinated interactions among the SN, DMN, and CEN. The SN, centred in the anterior insula and dorsal anterior cingulate cortex, detects behaviourally relevant internal and external stimuli and mediates switching between internally oriented and externally directed processing modes. The DMN supports autobiographical memory and self-referential processing, while the CEN enables cognitive control and goal-directed behaviour. Dynamic coordination among these networks integrates bodily signals, narrative identity, and behavioural agency, supporting coherent self-experience.

**Figure 2:**
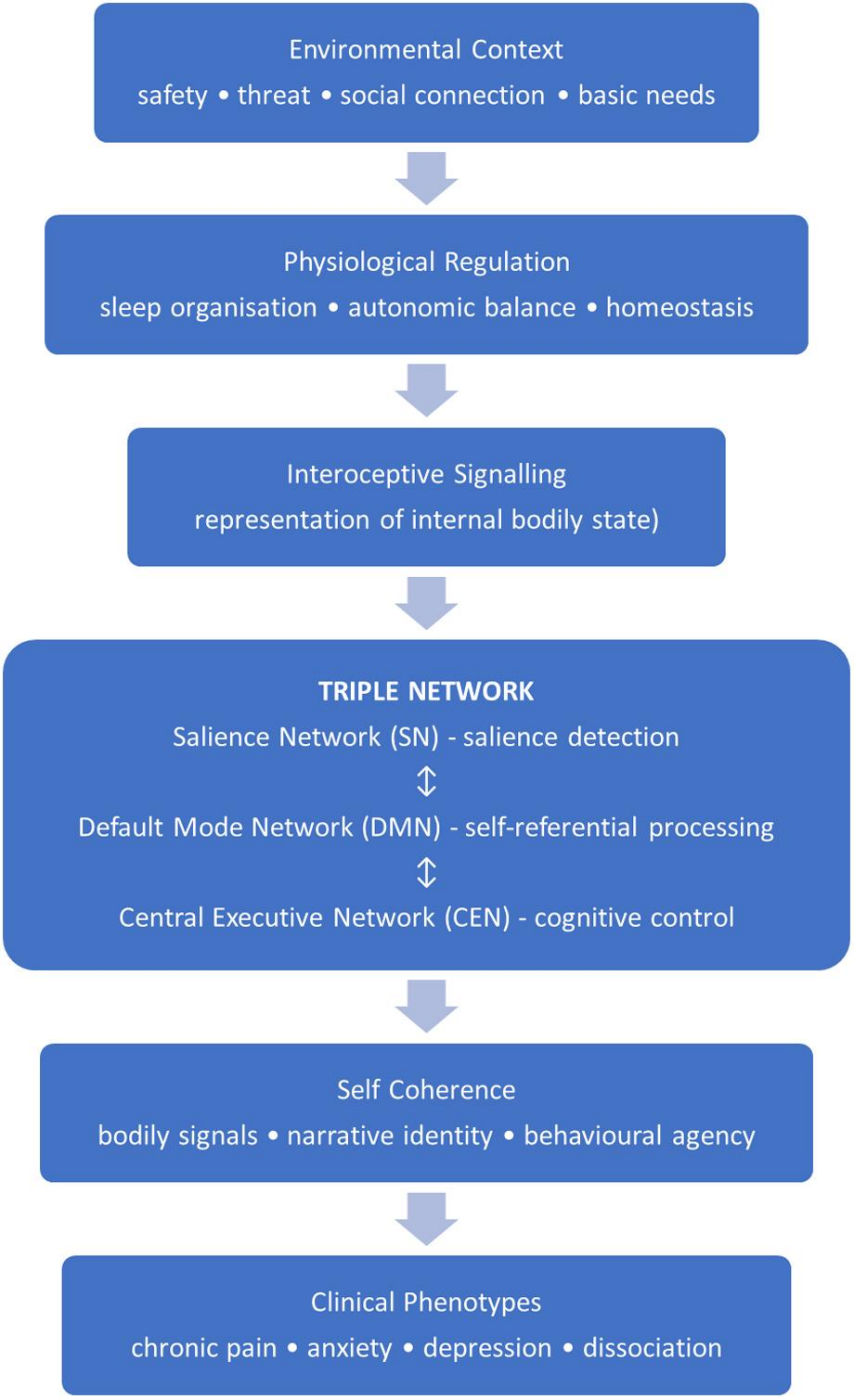
State-constrained triple-network model of self-organisation in chronic pain. Environmental conditions and basic regulatory needs influence organism-level physiological regulation, including sleep organisation, autonomic nervous system balance, and homeostatic bodily signalling. These physiological processes shape interoceptive signalling that informs salience processing within the brain. Interactions among the SN, DMN, and CEN integrate bodily signals, autobiographical processing, and executive control to support coherent self-experience. Disturbances of physiological regulation—such as those associated with trauma, chronic stress, or persistent nociceptive signalling—may bias salience processing toward bodily threat signals and destabilise coordination among these networks. This may lead to altered self-organisation and the emergence of clinical symptoms, including chronic pain.

**Figure 3:**
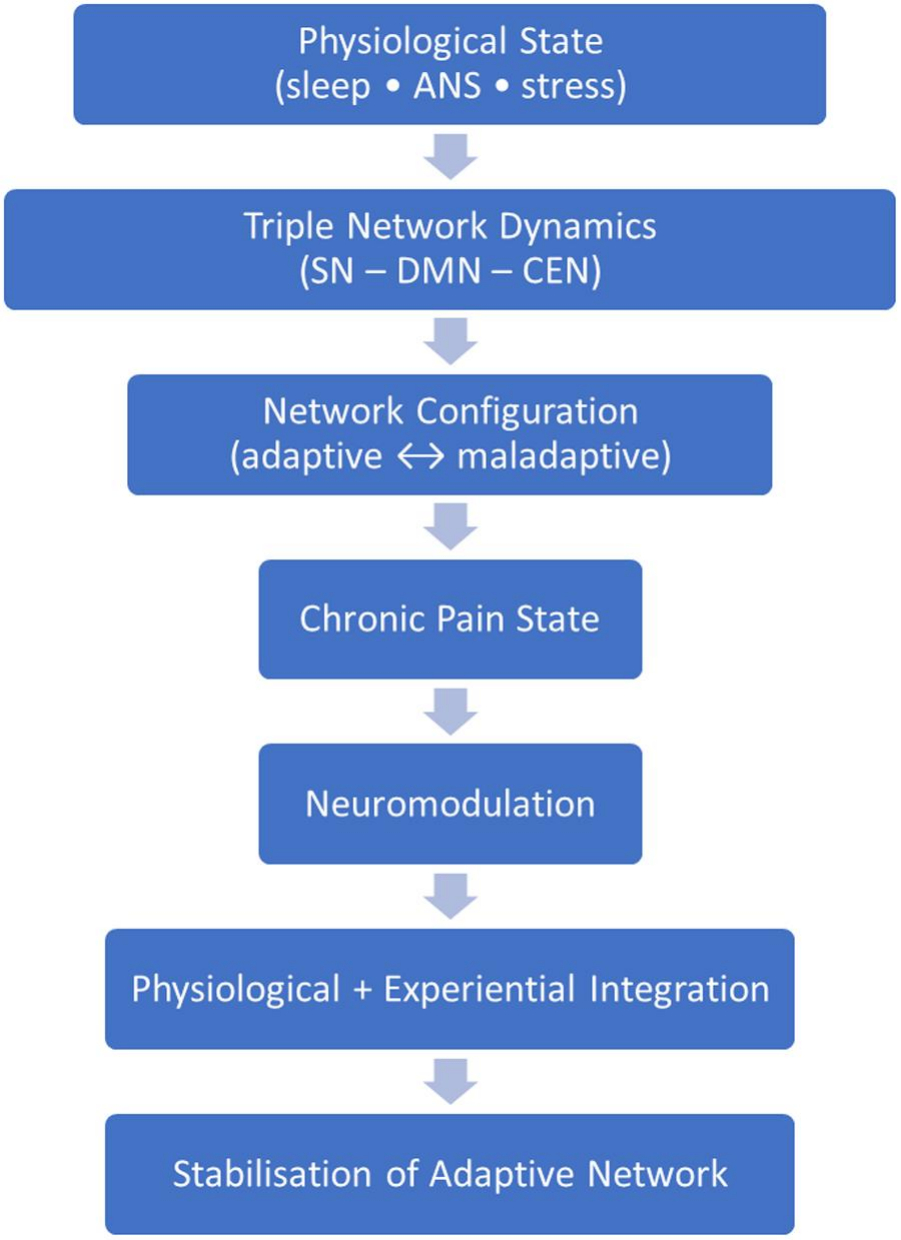
Dynamical model of triple-network attractor states and neuromodulatory intervention. Large-scale brain network activity can be conceptualised as evolving within a dynamical landscape of possible configurations. Under stable physiological regulation, interactions among the SN, DMN, and CEN networks remain flexible, allowing adaptive switching between internally oriented and externally directed processing. Persistent disturbances in physiological regulation, such as sleep disruption or autonomic hyperarousal, may shift network dynamics toward a stable maladaptive attractor configuration characterised by salience dominance and persistent attention to interoceptive pain signals. Neuromodulatory interventions such as repetitive transcranial magnetic stimulation (rTMS) may act as perturbations that increase network flexibility and destabilise maladaptive attractor states. Durable recovery may require concurrent restoration of physiological regulation and experiential integration to stabilise more adaptive network configurations.

Self-organisation refers to the emergence of stable patterns of neural activity from interactions among physiological regulation, large-scale network dynamics, and environmental conditions. In complex biological systems, stable behavioural and experiential states arise not from isolated neural mechanisms but from coordinated interactions among these regulatory processes over time (10, 11). The overall conceptual framework is illustrated in Figures 1–3.

## The triple-network architecture and the neural basis of self-organisation

Large-scale brain networks play a central role in coordinating cognition, emotion, and behaviour. Among these, the triple-network model, comprising the SN, DMN, and CEN, has been proposed as a core organisational architecture underlying adaptive brain function across a wide range of psychological processes (5). Rather than operating independently, these networks interact dynamically to integrate internal bodily signals, self-referential processing, and goal-directed behaviour.

The SN, anchored in the anterior insula and dorsal anterior cingulate cortex, is primarily involved in detecting behaviourally relevant internal and external stimuli (5). It integrates interoceptive signals with environmental information to determine the relative importance of incoming inputs and to prioritise behavioural responses (8, 12). In addition to detecting salient events, the SN plays a crucial regulatory role by mediating the dynamic switching between internally oriented and externally oriented modes of processing (5). Through this mechanism, it coordinates interactions between the DMN and CEN.

The DMN includes the medial prefrontal cortex, posterior cingulate cortex, and lateral parietal regions. This network is most active during internally directed cognitive processes, including autobiographical memory, future simulation, and self-referential thought (6, 13). The DMN is therefore often described as supporting aspects of the narrative self, providing continuity of personal experience across time through the integration of memory, identity, and meaning.

In contrast, the CEN, centred in the dorsolateral prefrontal cortex and posterior parietal cortex, supports cognitive control, working memory, and goal-directed behaviour (5). This network enables flexible regulation of attention and behaviour in response to changing environmental demands and contributes to the sense of agency and intentional control over actions.

Adaptive functioning depends not only on the integrity of each network but also on their coordinated interaction. The SN continuously evaluates incoming internal and external signals and facilitates switching between the internally oriented processes of the DMN and the externally focused cognitive control of the CEN (5). Through this dynamic coordination, the brain integrates bodily signals, autobiographical context, and executive control processes to support coherent perception, behaviour, and self-experience.

From this perspective, interactions among the SN, DMN, and CEN can be understood as forming a neural architecture that supports self-organisation, integrating bodily awareness, narrative identity, and cognitive agency within large-scale brain systems (10, 11). Disruptions in this coordinated system have been implicated across multiple neuropsychiatric conditions (5). In particular, growing evidence suggests that chronic pain is associated with altered connectivity and activity within these networks, including increased salience processing, changes in DMN dynamics, and reduced executive regulation (1–3). These findings raise the possibility that persistent pain may reflect disturbances in the large-scale network interactions that normally support the integration of bodily signals and self-related processing.

## Triple-Network dysregulation in chronic pain

Chronic pain is increasingly conceptualised as a disorder of large-scale brain network organisation rather than a direct reflection of ongoing peripheral nociceptive input (1, 2). Neuroimaging studies across multiple chronic pain conditions have consistently demonstrated alterations in functional connectivity within and between the SN, DMN, and CEN (3, 4). These findings suggest that persistent pain involves disturbances in the dynamic coordination of networks that normally integrate bodily signals, cognition, and self-related processing (2). Functional connectivity findings in chronic pain remain variable across studies, so altered connectivity should not be taken as evidence of a fixed pathological state, but as support for a testable network-level model.

One of the most consistent findings in chronic pain research is increased SN activity and altered salience processing. The anterior insula and dorsal anterior cingulate cortex, key nodes of the SN, are strongly involved in the perception and evaluation of interoceptive signals, including nociceptive input (8, 12). In individuals with chronic pain, these regions often show heightened activation and altered connectivity, suggesting that bodily signals related to pain may become persistently prioritised as behaviourally relevant (1, 7). This heightened salience weighting may bias attention toward internal bodily signals and contribute to the persistent awareness of pain.

Alterations are also frequently observed within the DMN, which is associated with self-referential processing and autobiographical thought (6, 13). Studies have shown that chronic pain is associated with disrupted DMN connectivity and abnormal interactions between the DMN and regions involved in pain processing (3, 14). These changes are thought to reflect the increasing integration of pain-related information into self-referential processing. Over time, persistent pain may become embedded within autobiographical narrative and identity, contributing to phenomena such as pain rumination and heightened self-focus on bodily symptoms.

The CEN also appears to be affected in chronic pain conditions. Reduced functional connectivity and altered activity within executive control regions have been reported in several chronic pain populations (1, 2). These changes may reflect diminished cognitive control over attention and reduced capacity to disengage from salient pain signals. As a result, the balance between internally oriented and externally oriented cognitive processing may become disrupted, with attention repeatedly captured by pain-related signals.

Importantly, these network alterations are not confined to individual networks but involve disrupted interactions between the SN, DMN, and CEN. The SN normally mediates switching between internally focused and externally directed processing by coordinating DMN and CEN activity (5). In chronic pain, persistent salience signalling related to bodily threat may bias this switching mechanism, favouring internally oriented processing and reinforcing pain-related attention and rumination (2).

From a network perspective, these changes may reflect the emergence of a stable configuration of network dynamics in which interoceptive pain signals become persistently prioritised and integrated into self-related processing (1, 3). This interpretation is consistent with proposals that chronic pain represents a shift in large-scale brain organisation rather than a purely sensory phenomenon (1). However, while current models describe patterns of network dysregulation associated with chronic pain, they provide limited explanation for why these network configurations persist over time.

Notably, chronic pain is also consistently associated with disturbances in physiological regulatory systems, including disrupted sleep architecture, autonomic imbalance, and altered interoceptive signalling (8, 9). These physiological processes are known to influence large-scale brain network dynamics (10, 11) but are rarely incorporated into prevailing network models of chronic pain. This raises the possibility that observed network alterations may not represent intrinsic network pathology but rather reflect network organisation occurring under persistent physiological regulatory constraints.

## Physiological regulation as a constraint on network dynamics

Although current models of chronic pain emphasise alterations in large-scale brain networks, these models often consider network dysfunction as primarily intrinsic to neural circuitry. However, growing evidence suggests that brain network dynamics are strongly influenced by organism-level physiological regulatory systems, including sleep organisation, autonomic nervous system balance, and interoceptive signalling. These systems continuously shape the internal milieu within which neural processing occurs and therefore represent important constraints on large-scale brain network organisation (10, 11).

Sleep plays a particularly important role in regulating brain network dynamics. Sleep architecture, including both slow-wave and rapid eye movement (REM) sleep, supports synaptic recalibration, emotional processing, and memory integration (15, 16). Disruptions in sleep have been shown to alter connectivity across multiple large-scale brain networks, including the DMN and SN (17). Importantly, chronic pain conditions are consistently associated with fragmented sleep, reduced sleep efficiency, and alterations in REM sleep organisation. Such disturbances may impair overnight recalibration of neural systems and contribute to the persistence of maladaptive network configurations.

Rapid eye movement (REM) sleep represents a physiological state characterised by coordinated changes in cortical activity and autonomic regulation that support large-scale neural recalibration. During REM sleep, interactions between limbic circuits, salience-related regions, and autonomic regulatory systems are thought to contribute to emotional memory integration and the processing of interoceptive signals (16, 17). Disturbances in REM sleep architecture are frequently reported in chronic pain conditions and may therefore disrupt the physiological conditions required for adaptive overnight reorganisation of large-scale brain networks. Within the present framework, REM sleep organisation may function both as a marker of physiological regulatory state and as a process through which large-scale network dynamics undergo adaptive recalibration across biological time (15).

Autonomic regulation represents a central mechanism through which physiological state influences large-scale brain network dynamics. The autonomic nervous system regulates the balance between sympathetic mobilisation and parasympathetic recovery, shaping interoceptive signalling and emotional processing (9). Regions central to the SN, including the anterior insula and anterior cingulate cortex, are closely linked to autonomic regulation and interoceptive representation (8, 12). Sustained sympathetic activation and reduced parasympathetic flexibility have been reported in several chronic pain conditions and may bias salience processing toward the detection of bodily threat signals. In this way, autonomic regulatory state may influence how interoceptive signals are prioritised within large-scale brain networks and contribute to persistent attention to pain-related bodily sensations.

These dynamics are also consistent with predictive processing frameworks, in which the brain continuously generates predictions about bodily states based on prior experience and incoming interoceptive signals (12, 18). Under conditions of persistent physiological dysregulation, predictive models of bodily threat may become reinforced, biasing salience processing toward pain-related signals and stabilising network configurations that prioritise interoceptive prediction errors associated with pain. In this sense, physiological regulatory state may influence how interoceptive predictions are generated and updated within large-scale brain networks. Sleep states, including REM sleep, are associated with coordinated changes in autonomic regulation and large-scale neural activity and may contribute to the overnight recalibration of these regulatory dynamics (16, 17).

Interoceptive signalling provides the neural representation of internal bodily states and plays a central role in shaping salience processing. Signals arising from visceral, metabolic, and nociceptive systems are integrated within cortical regions such as the anterior insula, which serves as a hub linking bodily state information to salience evaluation and higher-order cognitive processes (8, 18). Alterations in interoceptive processing have been described in both chronic pain and trauma-related conditions (12), suggesting that persistent changes in bodily signalling may influence how internal sensations are prioritised and interpreted within large-scale brain networks.

Direct evidence that physiological regulation causally drives specific triple-network transitions in chronic pain remains limited. Nevertheless, sleep disruption, autonomic dysregulation, and altered interoceptive signalling provide plausible and measurable constraints on large-scale network organisation (2, 8, 9, 12, 16, 17). These systems converge anatomically and functionally on salience-related regions, particularly the anterior insula and anterior cingulate/midcingulate cortex, providing a mechanism through which bodily regulatory state may bias attention toward pain-related signals. Current evidence therefore supports this as a testable physiological-network hypothesis, rather than an established causal pathway.

Taken together, these findings suggest that large-scale network dynamics operate within the constraints imposed by physiological regulatory states (10, 11). Sleep disruption, autonomic imbalance, and altered interoceptive signalling may create conditions in which salience processing becomes persistently biased toward bodily threat signals. Within such a regulatory context, the coordination of the SN, DMN, and CEN may shift toward configurations that prioritise internally oriented processing and reinforce persistent pain-related experience (2).

From this perspective, the network alterations observed in chronic pain may reflect not simply intrinsic neural dysfunction but rather adaptive network organisation occurring under conditions of sustained physiological dysregulation. This interpretation suggests that large-scale brain network configurations may be shaped by the broader physiological environment of the organism, raising the possibility that persistent pain reflects a stable pattern of network activity maintained by regulatory constraints (1). The following section introduces a conceptual model that integrates these physiological influences with triple-network dynamics to account for the persistence of chronic pain.

## A state-constrained triple-network model of self-organisation

The observations outlined above suggest that chronic pain may be better understood within a framework that integrates large-scale brain network dynamics with organism-level physiological regulation (1, 2). We therefore propose a state-constrained triple-network model of self-organisation, in which interactions among the SN, DMN, and CEN are continuously shaped by physiological regulatory states (5, 10, 11). Within this framework, coherent self-experience emerges from the coordinated integration of bodily signals, autobiographical processing, and executive control, while disruptions of physiological regulation can destabilise this coordination and lead to persistent clinical symptoms such as chronic pain.

In this model, the triple-network architecture functions as a regulatory processing system that integrates internal bodily signals and environmental information to generate perception, behaviour, and self-related experience (5). The SN plays a central role in evaluating interoceptive and environmental inputs, determining their behavioural relevance, and coordinating switching between internally oriented and externally oriented processing modes (5, 8). The DMN supports the narrative and autobiographical aspects of the self, integrating current experience with personal memory and identity (6, 13). The CEN provides cognitive control and supports the capacity for flexible behavioural responses and goal-directed action (5).

Coherent self-experience depends on synchronised interactions among these networks. Bodily signals arising through interoceptive pathways inform salience processing, which in turn regulates the balance between internally oriented self-referential processing within the DMN and externally directed cognitive control within the CEN (8, 12). Through this dynamic coordination, the brain maintains an integrated representation of bodily state, personal meaning, and behavioural agency.

However, the operation of this system does not occur in isolation from the broader physiological context of the organism. Sleep organisation, autonomic balance, and homeostatic bodily signalling continuously shape the conditions under which network interactions occur (9, 15, 16). When these physiological regulatory systems are stable, the triple-network architecture can flexibly coordinate attention, cognition, and self-related processing. In contrast, persistent disturbances in physiological regulation—such as those associated with trauma, chronic stress, or ongoing threat—may bias network dynamics toward configurations that prioritise internal bodily signals and threat-related processing (10, 11).

Under such conditions, interoceptive signals associated with pain may become persistently amplified within salience processing, leading to sustained prioritisation of bodily threat signals (8, 12). Over time, these signals may become increasingly integrated into self-referential processing within the DMN, contributing to the embedding of pain within autobiographical narrative and identity (3, 10). At the same time, impaired executive regulation may reduce the capacity to disengage attention from salient bodily sensations (2). The combined effect is a shift in large-scale network dynamics toward a stable configuration in which pain-related signals dominate internal processing.

Within the proposed model, chronic pain can therefore be conceptualised as a state-dependent configuration of triple-network dynamics, maintained by persistent physiological regulatory constraints (1, 10). Rather than representing a fixed structural abnormality, this configuration reflects the interaction between network processing and the organism's regulatory state. Disturbances of sleep, autonomic imbalance, and altered interoceptive signalling may stabilise network patterns that prioritise bodily threat signals and reinforce self-referential pain processing (9, 16).

This perspective provides a framework for understanding how chronic pain may persist even in the absence of ongoing peripheral pathology (1). It also links network neuroscience with the phenomenology of self-experience, suggesting that persistent pain reflects a disruption in the integration of bodily signals, narrative self-processing, and cognitive control. By situating triple-network dynamics within the broader context of physiological regulation, the state-constrained model offers a multilevel explanation for the persistence and variability of chronic pain states.

In the following section, we consider how these dynamics may be interpreted within a broader dynamical systems framework and explore the implications for neuromodulatory interventions aimed at modifying maladaptive network configurations (10, 11).

## Dynamical interpretation and implications for neuromodulation

The state-constrained triple-network model can also be interpreted within a broader dynamical systems framework, in which large-scale brain activity evolves over time within a landscape of possible network configurations (10, 11). From this perspective, brain networks do not operate in fixed states but instead transition between patterns of activity depending on internal and external conditions. These patterns can be conceptualised as attractor states, representing relatively stable configurations of network dynamics that the system tends to occupy (10).

Under normal physiological conditions, interactions among the SN, DMN, and CEN remain flexible, allowing the brain to dynamically shift between internally oriented processing, external task engagement, and responses to salient environmental stimuli (5). Sleep organisation, autonomic balance, and interoceptive signalling help maintain this flexibility by supporting physiological regulation and neural recalibration across biological time (9, 15, 16). These processes enable the system to adaptively transition between network configurations in response to changing environmental and bodily demands (10, 11).

However, when physiological regulation becomes persistently disrupted—such as through chronic stress, trauma, or sustained nociceptive signalling—the dynamical landscape of network interactions may shift. In such cases, the brain may settle into a stable but maladaptive attractor configuration in which salience processing becomes persistently biased toward bodily threat signals (1, 2). Within this configuration, interoceptive signals associated with pain may be continually prioritised by the SN, reinforced through self-referential processing in the DMN, and insufficiently modulated by executive control systems (3). The result is a persistent experiential state in which pain remains highly salient and difficult to disengage from.

This dynamical interpretation helps explain several clinical features of chronic pain. Persistent pain states often display stability over time, resistance to conventional treatments, and fluctuating symptom intensity depending on physiological conditions such as sleep quality or stress (1). These features are consistent with the behaviour of systems operating within attractor dynamics, where transitions out of a stable configuration may require sufficient perturbation to shift the system into an alternative state (10, 11).

Within this framework, neuromodulatory interventions such as repetitive transcranial magnetic stimulation (rTMS) may act as perturbative inputs capable of temporarily destabilising maladaptive network configurations. By modulating activity in key nodes of the triple-network architecture—particularly within prefrontal or motor cortical regions—rTMS may increase network flexibility and facilitate transitions away from persistent pain-related attractor states (19, 20). Emerging approaches that use connectome-guided targeting strategies aim to influence network-level interactions rather than isolated cortical regions, further supporting this network-based perspective (19, 20).

In chronic pain, rTMS is most commonly targeted to the precentral cortex, traditionally described as M1 stimulation (21, 22). Its analgesic effects, however, are unlikely to reflect local motor cortex modulation alone. Through distributed cortico-subcortical connections, precentral stimulation may influence pain-modulatory systems and thereby perturb broader sensorimotor, SN, DMN, and CEN dynamics within persistent pain-related network states.

However, if the broader physiological conditions that maintain the maladaptive attractor configuration remain unchanged, the system may gradually return to its previous state. From a dynamical perspective, durable clinical change may therefore require not only direct modulation of neural network activity, but also restoration of the physiological regulatory processes that shape the system's operating environment (9, 16). Improvements in sleep architecture, autonomic flexibility, and interoceptive regulation may help stabilise more adaptive network configurations following neuromodulatory intervention.

Viewed in this way, neuromodulation may function primarily as a mechanism for increasing network flexibility, enabling the system to transition away from entrenched attractor states (10, 11). The consolidation of more adaptive patterns of network organisation may then depend on the restoration of physiological regulation and the integration of new experiential and behavioural patterns over time. This perspective highlights the importance of combining neuromodulatory interventions with approaches that address physiological regulation and psychological integration in the treatment of chronic pain.

## Predictions and future research

The state-constrained triple-network model generates several testable predictions regarding the relationship between physiological regulation, large-scale brain network dynamics, and the persistence of chronic pain. By situating triple-network interactions within organism-level regulatory systems, the model suggests that alterations in sleep, autonomic balance, and interoceptive signalling should influence both network organisation and clinical symptom expression (5, 10, 11).

First, the model predicts that physiological regulatory states should correlate with patterns of triple-network connectivity. For example, disruptions in sleep architecture—particularly reductions in slow-wave and REM sleep—may be associated with increased SN activity and stronger coupling between salience and DMNs (16, 17). Conversely, improvements in sleep quality may be associated with increased network flexibility and reduced salience dominance. Longitudinal neuroimaging studies examining changes in network connectivity before and after sleep interventions may therefore provide important insights into the relationship between physiological regulation and chronic pain states (10, 11).

Second, autonomic nervous system regulation may play a measurable role in shaping network dynamics. Measures of autonomic flexibility, such as heart rate variability, may correlate with the capacity of the SN to appropriately switch between internally oriented and externally oriented processing modes (5, 9). Reduced autonomic flexibility may therefore be associated with persistent salience weighting of interoceptive signals and increased attention to pain-related bodily sensations (8, 12).

Third, the model predicts that neuromodulatory interventions such as rTMS may produce greater and more durable clinical benefit when physiological regulatory conditions are favourable. For example, individuals with more stable sleep patterns or greater autonomic flexibility may show larger or more sustained responses to network-targeted neuromodulation. Conversely, persistent physiological dysregulation may limit the durability of neuromodulatory effects by maintaining the regulatory conditions that stabilise maladaptive network configurations (19, 20).

Fourth, the model suggests that chronic pain may involve increased coupling between interoceptive processing regions and networks involved in self-referential processing, particularly the SN and DMN. Future studies combining measures of interoceptive accuracy, network connectivity, and clinical pain measures may help clarify the mechanisms through which bodily signals become integrated into persistent pain experience (6, 8, 12).

Finally, the model highlights the potential importance of multimodal treatment approaches that address both neural network dynamics and physiological regulation. Interventions targeting sleep restoration, autonomic regulation, and psychological integration may interact with neuromodulatory approaches to promote more durable shifts in network organisation. Future research integrating neuroimaging, physiological monitoring, and clinical outcomes will be important in evaluating these interactions (9, 16).

Individual differences are likely to shape how pain-related network states become stabilised and how readily they respond to treatment. Psychological vulnerability, trauma history, affective symptoms, pain-related fear, and the degree to which pain becomes incorporated into self-experience may influence SN and DMN weighting of pain signals. Biological and clinical factors, including sleep phenotype, autonomic flexibility, medication exposure, and pain duration, may further affect the stability of maladaptive network configurations and the capacity for neuromodulatory or multimodal interventions to shift the system toward more adaptive states (1–3, 9).

Taken together, this framework suggests that persistent pain may represent a state-constrained pattern of self-organisation arising from interactions among physiological regulation, metastable large-scale brain network dynamics, and ongoing experiential processes (10, 11).

## Conclusion

Chronic pain is increasingly recognised as a disorder involving large-scale brain network organisation rather than a simple consequence of persistent nociceptive input (1, 2). Research across multiple chronic pain conditions has demonstrated alterations in the SN, DMN, and CEN, suggesting that disturbances in the coordination of these systems contribute to the persistence of pain (3, 4). However, prevailing models typically interpret these findings as intrinsic network dysfunction and provide limited explanation for why these configurations emerge or remain stable over time.

In this paper, we proposed a state-constrained triple-network model of self-organisation that situates network dynamics within the broader physiological regulatory context of the organism (5, 10, 11). In this framework, interactions among the SN, DMN, and CEN support the integration of bodily signals, autobiographical processing, and cognitive control that together contribute to coherent self-experience. Chronic pain may arise when disturbances in physiological regulation—including disrupted sleep, autonomic imbalance, and altered interoceptive signalling—bias salience processing toward bodily threat signals and stabilise network configurations that reinforce persistent pain-related experience (8, 9, 16).

From this perspective, the network alterations observed in chronic pain may reflect adaptive but maladaptive stabilisation of network dynamics under conditions of regulatory constraint, rather than fixed neural pathology. This interpretation provides a potential explanation for the persistence, variability, and treatment resistance often observed in chronic pain conditions.

The model also has implications for treatment. Neuromodulatory approaches such as repetitive transcranial magnetic stimulation may act as perturbations capable of increasing network flexibility, but durable clinical improvement may depend on concurrent restoration of physiological regulatory processes and the integration of new experiential patterns over time (19, 20).

By linking physiological regulation, large-scale brain network dynamics, and the phenomenology of self-experience, the state-constrained triple-network framework offers a multilevel perspective on chronic pain and highlights potential avenues for future research and integrated treatment strategies.

## Data Availability

The original contributions presented in the study are included in the article/Supplementary Material, further inquiries can be directed to the corresponding author.
